# Feed-forward visual processing suffices for coarse localization but fine-grained localization in an attention-demanding context needs feedback processing

**DOI:** 10.1371/journal.pone.0223166

**Published:** 2019-09-26

**Authors:** Sang-Ah Yoo, John K. Tsotsos, Mazyar Fallah

**Affiliations:** 1 Department of Psychology, York University, Toronto, ON, Canada; 2 Centre for Vision Research, York University, Toronto, ON, Canada; 3 Active and Attentive Vision Laboratory, Department of Electrical Engineering and Computer Science, York University, Toronto, ON, Canada; 4 Visual Perception and Attention Laboratory, School of Kinesiology and Health Science, York University, Toronto, ON, Canada; Universite Toulouse III - Paul Sabatier, FRANCE

## Abstract

It is well known that simple visual tasks, such as object detection or categorization, can be performed within a short period of time, suggesting the sufficiency of feed-forward visual processing. However, more complex visual tasks, such as fine-grained localization may require high-resolution information available at the early processing levels in the visual hierarchy. To access this information using a top-down approach, feedback processing would need to traverse several stages in the visual hierarchy and each step in this traversal takes processing time. In the present study, we compared the processing time required to complete object categorization and localization by varying presentation duration and complexity of natural scene stimuli. We hypothesized that performance would be asymptotic at shorter presentation durations when feed-forward processing suffices for visual tasks, whereas performance would gradually improve as images are presented longer if the tasks rely on feedback processing. In Experiment 1, where simple images were presented, both object categorization and localization performance sharply improved until 100 ms of presentation then it leveled off. These results are a replication of previously reported rapid categorization effects but they do not support the role of feedback processing in localization tasks, indicating that feed-forward processing enables coarse localization in relatively simple visual scenes. In Experiment 2, the same tasks were performed but more attention-demanding and ecologically valid images were used as stimuli. Unlike in Experiment 1, both object categorization performance and localization precision gradually improved as stimulus presentation duration became longer. This finding suggests that complex visual tasks that require visual scrutiny call for top-down feedback processing.

## Introduction

The human visual system is known to be very rapid and efficient at analyzing some types of visual information. People can determine whether a briefly flashed image contains a depiction of a certain object category and categorization performance still holds even if another visual pattern immediately follows the target image by backward-masking or rapid serial visual presentation (RSVP) [1–10]. Since object categorization is performed within a very short period of time, this process is thought to rely on feed-forward visual processing [11,12]. Besides behavioral evidence, electroencephalographic activity demonstrates the same point. ERP analysis revealed a divergence in voltage between category-present and category-absent trials that developed after 150 ms of stimulus onset [1,6,13]. Studies using classifier-based readout techniques also demonstrated that information about object category and identity can be decoded from human temporal cortex and macaque inferior temporal area (IT) as early as 100 ms after stimulus onset, suggesting that hierarchical feed-forward processing is sufficient for rapid object categorization [14–16].

The architecture of the visual system also impacts how object location information is represented. It is well-established that the ventral pathway of the visual system is structured as a layered pipeline where each area, from the retina to the temporal cortex, features increasing receptive field (RF) sizes (nicely documented in [17]) and fewer representational columns, as a visual signal traverses the pipeline in its feed-forward journey. All neurons within each area receive converging input from the previous area organized in a spatially-limited RF and provide diverging input to many neurons in a reciprocal spatially-limited manner in the next area (see [18] for neuroanatomical and [19] for computational discussions). This structure blurs location precision, e.g. the Blurring Problem [20]. Thus, the precision of location representation is necessarily different for each area of this pipeline, with areas least affected by blurring (earliest) having the most precise location representation and those most affected by blurring (highest) the worst. Recent neuroimaging research supports that high-order object areas represent coarse object locations [21–29]. Coarse location information in higher visual areas would be sufficient if a visual task asks for approximate object location, and it could be accessed rapidly. For instance, saccades toward the visual hemifield where the animal target was presented could be initiated in as little as 120 ms [30] and saccadic latency was even shorter when human faces were the target [31], meaning that the coarse target location was necessarily processed before the minimum saccadic latency.

**Fig 1:**
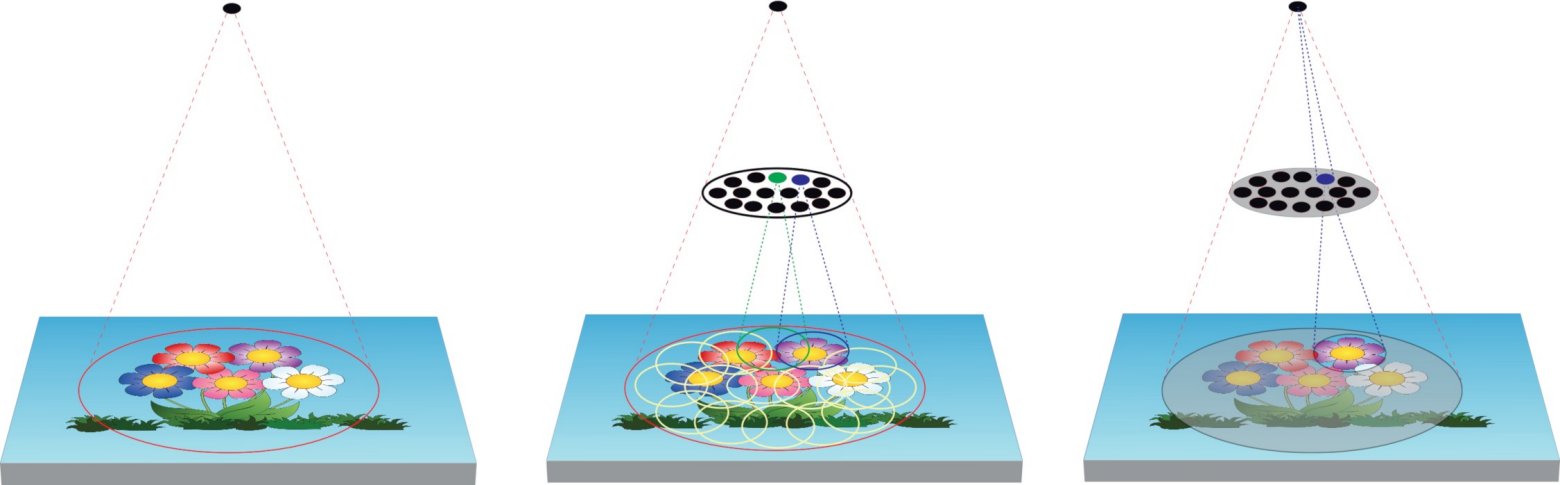
A schematic illustration of top-down feedback processing in visual hierarchy. (Leftmost) A hypothetical neuron whose RF sees the whole bunch of flowers. This neuron does not selectively respond to individual flowers. For example, if that neuron were trained to prefer a single flower with yellow center and purple petals, it would respond only partially because of all the other flowers in the same RF [42]. (Middle) Each neuron in an intermediate layer has a smaller RF. If the neuron in green were tuned to the flower with yellow center and purple petals, it would respond only partially. On the other hand, the neuron in blue with the same tuning property would respond maximally. All the other neurons regardless of what kind of flower they might be tuned to, would also respond partially or fully, each to their own tuning. (Rightmost) The neuron in the top layer receives all inputs from the intermediate neurons. If precise localization of the flower with yellow center and purple petals is needed, the neuron in the top layer could selectively receive input from the intermediate neuron which maximally respond to the target (neuron in blue). In ST, it is achieved by suppressing the inputs coming from all the other neurons in a top-down manner (i.e., attentional surround suppression). This top-down inhibition process operates layer by layer until it reaches the earliest one where the finest location information required for the task is represented.

On the other hand, more precise object localization would require access to early areas of the visual processing hierarchy since that is where this information is best represented. These early areas cannot represent a complex object as a whole, while higher visual areas see an entire object, but they encode only coarse location information. Thus, higher visual areas need to access fine-grained location information in early visual areas to determine an object’s precise location (e.g., Selective Tuning model; [32–34]). The Selective Tuning model suggested that different types of visual recognition tasks would go through different processing steps across the visual hierarchy, and that the amount of time it takes to achieve the task indicates the stage of recognition. For example, a single feed-forward pass would suffice for simple discrimination or categorization tasks (in ST, Convergence Binding, [34]) and its time course would be consistent with the time courses that the previous studies on ultra-rapid object categorization have demonstrated [1,6,13]. However, if a task requires detailed feature binding or localization, subsequent top-down feedback signals should reach earlier areas to recover ambiguous location information thereby increasing processing time (in ST, Full Recurrence Binding). Even though the visual hierarchy pipeline does indeed have both short-range and long-range feedback connections between several areas, it necessarily takes additional time to use those connections for access to early areas. This is so because the nature of connectivity just described imposes crosstalk (the Crosstalk Problem, [20]); multiple converging signals interfere and corrupt each other. If there is no mechanism to select one signal over another, the blurring of location and ambiguity of category information would persist. The choice is whether the visual system simply decodes a corrupted signal or actively attempts to clean the signal before its interpretation. The Selective Tuning model takes the latter position and provides such a mechanism that combines competitive attentional selection with suppression of interference that progresses in a top-down manner from higher to lower cortical areas [20,32,35,36]. This progression is responsible for the additional time observed when more precise location information is required by a given visual task. Fig 1 illustrates the hierarchical structure of neuronal RFs and a schematic feedback processing suggested by ST. Here, feedback processing means the top-down, attention-mediated signal that is temporally and functionally dissociated from rapid local recurrent processing within the ventral visual stream [35–40]. Evans and Treisman [41] asked participants to detect a target object in a rapid serial visual presentation (RSVP, each stimulus was presented for 75 ms) sequence and then report its identity and spatial location (left, center or right side of the image). Similar to ST’s claim, their participants could not report even the rough position of a given object in the image, although its detection was successful. It implies that localization requires top-down feedback processing, thus, additional processing time.

In the present study, we examined whether feed-forward processing is sufficient for object localization or whether the subsequent top-down, attention-mediated feedback processing should be involved in localization as ST predicts. We conducted an animal detection task as a replication of the previously reported ultra-rapid visual categorization task [1,6,8,13,43] and a novel localization task that asks participants to report the spatial location of a given animal feature (e.g., “Where is the tail?). Critically, we constrained the presentation durations of images so that we can determine how much processing time is needed to perform each type of task. We hypothesized that if feed-forward signals enable both visual tasks, performance would level off from relatively shorter presentation durations, whereas performance would continuously improve as presentation duration becomes longer if feedback signals enhance recognition processes. Stimulus complexity varied between Experiments 1 and 2.

## Experiment 1

In Experiment 1, each participant performed animal detection tasks to replicate the previous findings and animal feature localization tasks to examine the role of feedback processing in object localization. In the feature localization tasks, participants reported the location of a certain feature by clicking on a screen location using a mouse. Presentation duration of the stimuli varied to manipulate the visual processing time.

### Materials and methods

#### Participants

Forty-eight naïve participants (12 men, 36 women), between the ages of 17 and 39 years completed the experiment. 24 participants performed the *before* condition and the other 24 participants performed the *after* condition where a task question was shown before or after stimulus presentation, respectively. They had normal or corrected-to-normal vision and their color vision was also intact. Informed written consent was obtained from all participants. Participants, who were recruited from the Undergraduate Research Participants Pool of York University, received course credit for their study participation and the other participants were paid $15 CAD. The research was approved by York University’s Human Participants Review Committee. According to the human research guidelines of York University, older minor age participants (e.g. 17 years old) do not require parental consent to participate in this research, as it fell under the category of minimal risk research.

#### Apparatus and stimuli

Experiments were conducted in a dark room. Participants sat 57 cm from a CRT monitor (21” View Sonic G225f, 1280 x 1024, 85 Hz) and their heads were stabilized on a head and chin rest (Headspot, UHCOtech, Houston, TX). Participants wore an infrared eye tracker (Eyelink II, SR Research, 500 Hz, Mississauga, ON, Canada) monitoring the left eye position. Experimental control was maintained by Presentation (Neurobehavioral Systems, Berkeley, CA).

We used 400 images (200 animal-present, 200 animal-absent) that are the same images used in [6] or similar images collected from the Internet. The images subtended roughly 16° visual angle in width and 22° visual angle in height. Animal targets included mammals, birds, insects, fish, amphibians, and reptiles. Only real animals were counted as targets and humans were not categorized as animals in this experiment. Each animal-present image contained one animal (humans were not presented together), located in the central area of the image. The distractor images included natural landscapes (e.g., mountains, forests, lakes, and oceans), cityscapes, plants, buildings, and other man-made objects. Each image had a corresponding masking stimulus created by randomly scrambling pixels from the original image. Stimuli and data of all experiments are available at https://osf.io/qy5fm/.

#### Procedure

**Fig 2:**
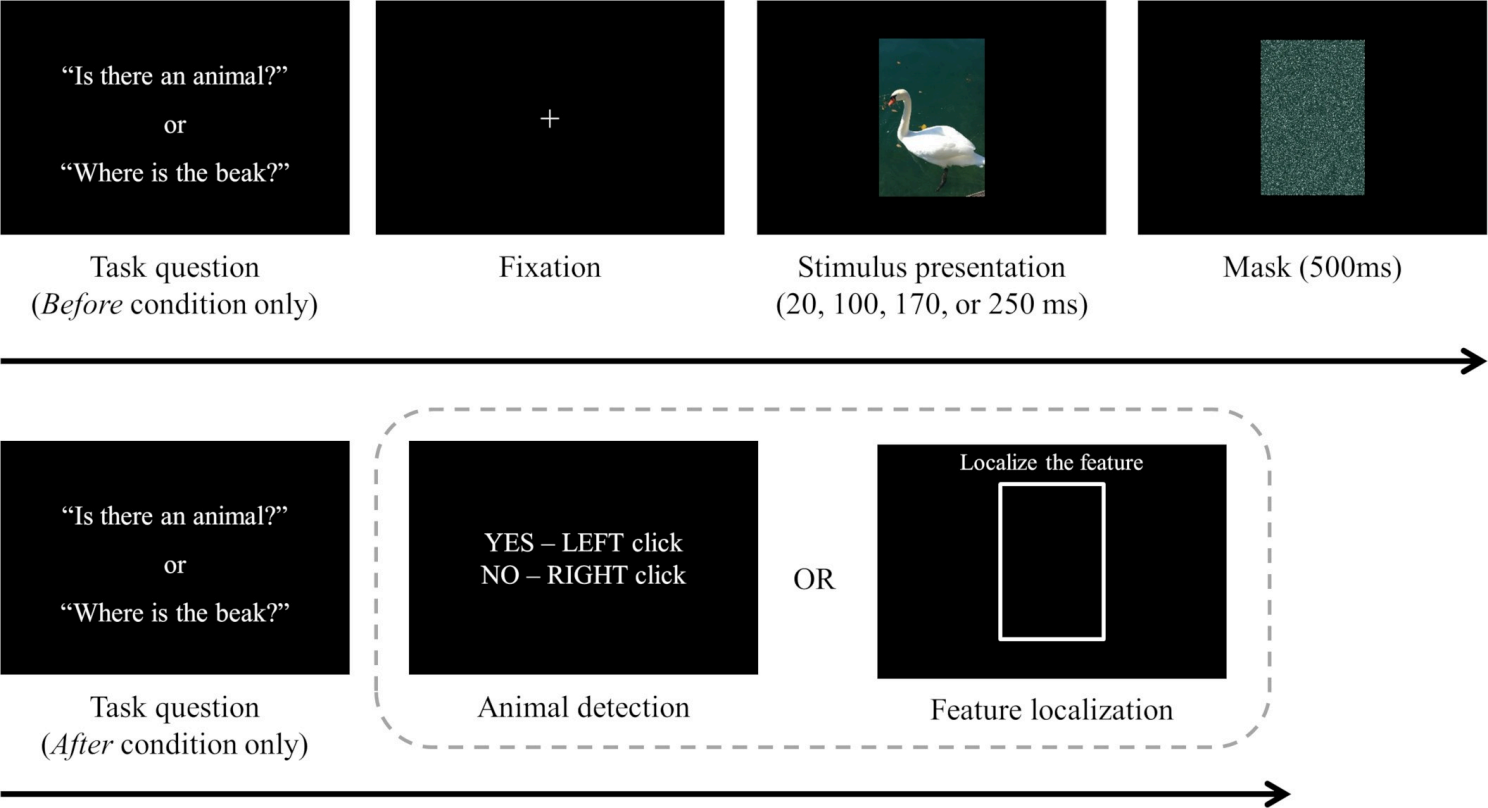
Procedure of Experiment 1. Participants performed randomly interleaved animal detection and feature localization trials. The stimulus was presented for 20, 100, 170, or 250 ms and it was immediately masked. A task question was shown either before or after stimulus presentation depending on which condition a participant was assigned to. Participants reported the presence of the animal target or localized a given animal feature using a mouse. The animal image in this figure is similar but not identical to the tested images (for illustrative purposes only).

Fig 2 depicts the procedure of Experiment 1. In the *before* condition, participants viewed a task question first. If the task of a given trial was animal detection, the question was “is there an animal?” or if the task was feature localization, participants were asked to localize a certain feature of an animal (e.g., “where is the beak?”). Animal detection and feature localization trials were randomly interleaved. Participants had to look at the fixation cross appearing in the center of the screen before stimulus presentation. When their eyes were fixated, an image could be presented for 20, 100, 170, or 250 ms and then immediately masked for 500 ms. Each image was presented only once throughout the experiment. Participants who were assigned to the *after* condition saw the task question after stimulus presentation. If the task was animal detection, participants clicked the left or right mouse button to report whether the animal target was present or absent, respectively. If the task was feature localization, a white reference frame that was equally-sized with the images was presented in the center of the screen. Participants were instructed to click on the location within that frame that corresponded to the location of the target feature in the test image. For both tasks, there was no time constraint for responses. Each participant completed 80 animal detection trials (40 animal-present and 40 animal-absent trials) and 80 feature localization trials (all animal-present) in a single session.

### Results

#### Animal detection

**Fig 3:**
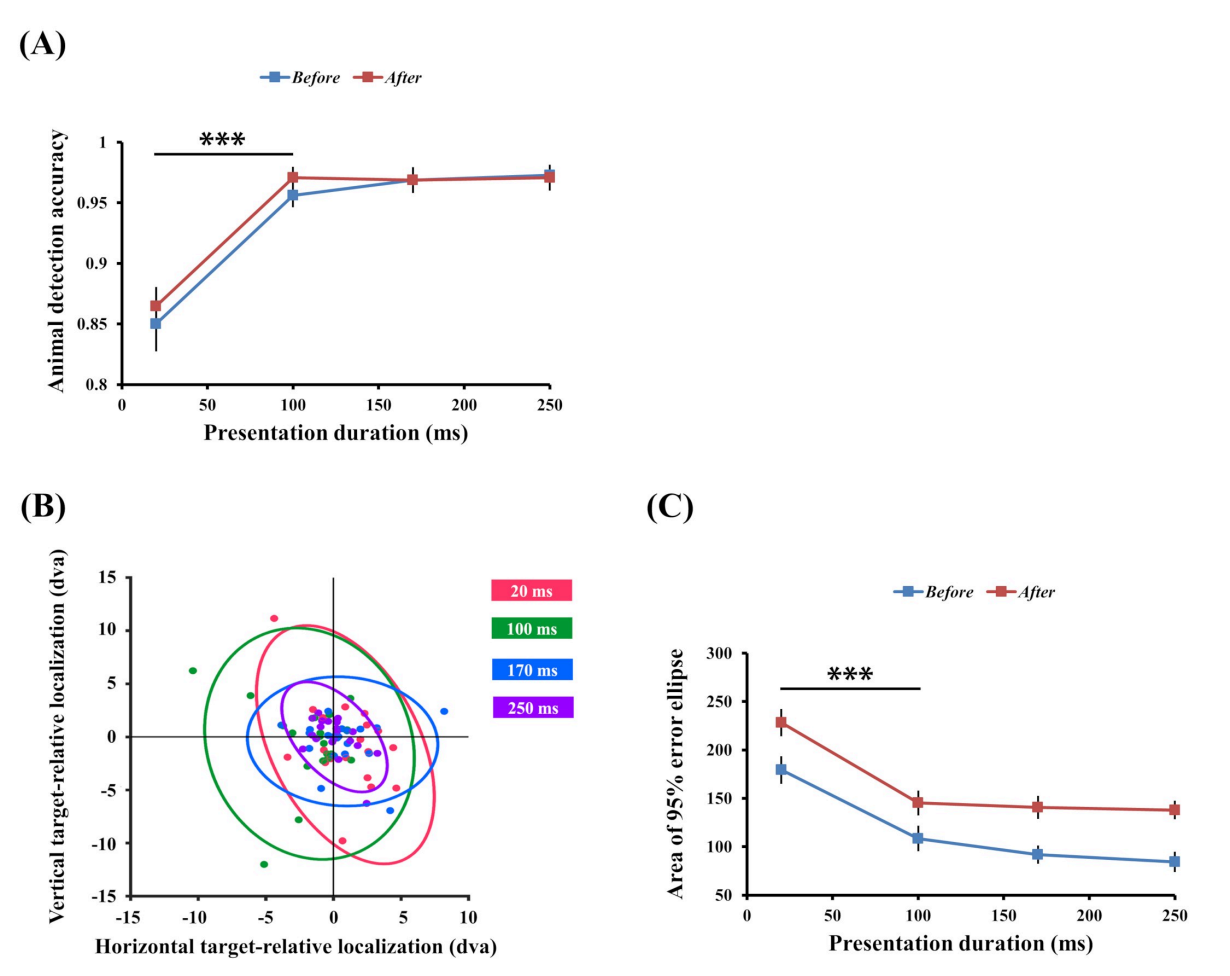
Experiment 1 results. (A) Animal detection accuracy improved between 20 ms and 100 ms but it plateaued after 100 ms. This result did not vary by the timing of the task questions (before or after stimulus presentation). (B) An example of 95% localization error ellipses for each presentation duration. The centroids of the target features were normalized to (0, 0) and each colored dot indicates the participant’s localization response relative to the target centroid. (C) Feature localization error was reduced between 20 ms and 100 ms but localization performance did not significantly change at longer presentation durations. Mean localization performance was better in the *before* condition but the performance pattern across different presentation durations was the same in both the *before* and *after* conditions. Error bars indicate SEM. *** *p* < .001.

Mean animal detection accuracy was significantly affected by stimulus presentation duration (Fig 3A; Greenhouse-Geisser correction (ε = .668); *F*(2, 92.18) = 56.372, *p* < .001), but the main effect of the *before-after* conditions (*F*(1, 46) = .274, *p* = .603) and the interaction between presentation duration and the *before-after* conditions were not significant (*F*(2, 92.18) = .376, *p* = .688). Viewing the task question prior to stimulus presentation is not necessary for accurate animal detection because even participants assigned to the *after* condition could judge the presence/absence of an animal easily once they saw an image. In both *before* and *after* conditions, animal detection accuracy sharply improved between 20 ms to 100 ms (all *p*s < .001) and then it leveled off (all *p*s > .05). We do not provide the reaction time (RT) data here because there is no comparable starting point for RTs between the *before* and *after* conditions. While RTs in the *before* condition can be measured after mask offset, RTs in the *after* condition unavoidably vary depending on how quickly participants read and understand task questions after viewing the stimulus. In addition, there was no time constraint for responses and the participants were not required to make speeded responses. The results suggest that detecting the presence of an animal can be done within a very short period of time in this image set, replicating ultra-rapid visual categorization [1,3,4,6].

#### Feature localization

To quantify the localization precision, we measured the area of the 95% error ellipse of participants’ localization responses relative to the normalized target centroids [44]. Fig 3B shows one participant’s localization performance for different presentation durations and the corresponding error ellipses. The ellipse size indicates localization error, thus, a smaller ellipse means higher localization precision. Mean localization error was significantly modulated by presentation duration (Fig 3C; Greenhouse-Geisser correction (ε = .755); *F*(2,27, 104.21) = 38.82, *p* < .001) and by the *before-after* conditions (*F*(1, 46) = 14.39, *p* < .001). Post-hoc multiple comparisons with Bonferroni correction showed that the localization error was lower in the *before* condition than in the *after* condition (M_diff_ = -47.01, SE = 12.39, *p* < .001). The interaction between presentation duration and the *before-after* condition was not significant (*F*(2.27, 104.21) = .27, *p* = .791). As this null interaction indicated, localization performance patterns in both *before* and *after* conditions were almost equal across different presentation durations. In both conditions, localization was more precise when the presentation duration was extended from 20 ms to 100 ms (all *p*s < .001) but performance did not improve after 100 ms (all *p*s > .05). Therefore, similar to the animal detection results, feature localization seemed to be completed very rapidly and the current results do not support the idea that feedback processing improves localization precision.

## Experiment 2

Experiment 1 replicated prior rapid animal detection results and also suggested that feature localization may be accomplished within the feed-forward sweep. This seems to place our central hypothesis in doubt, namely, that feedback is required for localization. One possibility for this is that the animal-present images in Experiment 1 might have been too simple so that the result of this experiment did not represent detailed localization which demands full top-down processing [34,45]. Each image contained only one animal that occupied most of the central area of the image, and its background was also very simple so that the target animal was well segregated from the background. Hence, participants might be able to extract the target animal’s layout easily and guess the location of a target feature based on this gist rather than carefully localizing the feature by attention-demanding feature binding and distractor suppression. For instance, if one sees the contour of a bird’s head then s/he could guess the relative location of a certain feature (e.g., beak) on its head. In other words, the cross-talk or interference within the visual hierarchy that would necessitate top-down feedback for correction is not so a great problem for images where there is no real visual conflict. A similar situation occurs in visual search where targets are well-differentiated from distractors (i.e., pop-out) and no focused attention is required. To control for this potential confounding factor, we conducted the same experiments with different stimuli. We used more complex animal-present images that contained more than one animal or (an) animal(s) with human(s), embedded in a complex background. In these images, the segregation between the target and background would not immediately occur, hence, more precise localization processes would be required. These new images should be more similar to the visual scenes we confront in our daily life. The masking stimuli in Experiment 2 were random polygons in random colors so that they were unrelated to the original images.

### Materials and methods

#### Participants

Twenty-four naïve participants (9 men, 15 women), between the ages of 17 and 34 years completed the experiment. They had normal or corrected-to-normal vision and their color vision was also intact. Informed written consent was obtained from all participants. Participants who were recruited from the Undergraduate Research Participants Pool of York University received course credit for their study participation while other participants were paid $15 CAD. The research was approved by York University’s Human Participants Review Committee. According to the human research guidelines of York University, older minor age participants (e.g. 17 years old) do not require parental consent to participate in this research, as it fell under the category of minimal risk research.

#### Apparatus and stimuli

The apparatus was the same as in Experiment 1. We used 420 new images (210 animal-present, 210 animal-absent) collected from the Internet and selected from the MS COCO dataset (http://mscoco.org, [46]). The images subtended roughly 20° visual angle in width and 13° visual angle in height. In animal-present images, animal targets were mostly mammals and birds that live in groups or live with humans. Each image contained more than one animal or (an) animal(s) with human(s), and humans were again not categorized as animals. The size and location of the target animal randomly varied across different images, but it was not too small or too peripheral. The backgrounds of the images included natural landscapes, cityscapes, outdoor and indoor scenes. The animal-absent distractor images were also drawn from similar scene categories. Masking stimuli were created in MATLAB (The MathWorks, Inc.) with the Psychophysics Toolbox [47,48]. They consisted of multiple random polygons that had different sizes, shapes, and colors.

#### Procedure

The experimental procedure was the same as in Experiment 1, except that the task question was always shown before stimulus presentation and we changed presentation durations. We did not test both the *before* and *after* conditions because in Experiment 1, these conditions did not affect the performance patterns across different presentation durations in the animal detection and feature localization tasks. When an image contained multiple animals, the task question was specific to one of these animals so that participants would not be confused (e.g., “Where is the muzzle of the black-and-white cow?”). An image could be presented for 25, 50, 75, 100, 170, 250, or 300 ms. Each participant completed 140 animal detection trials (70 animal-present and 70 animal-absent trials) and 140 feature localization trials (all animal-present), and these trials were randomly interleaved.

### Results

#### Animal detection

**Fig 4:**
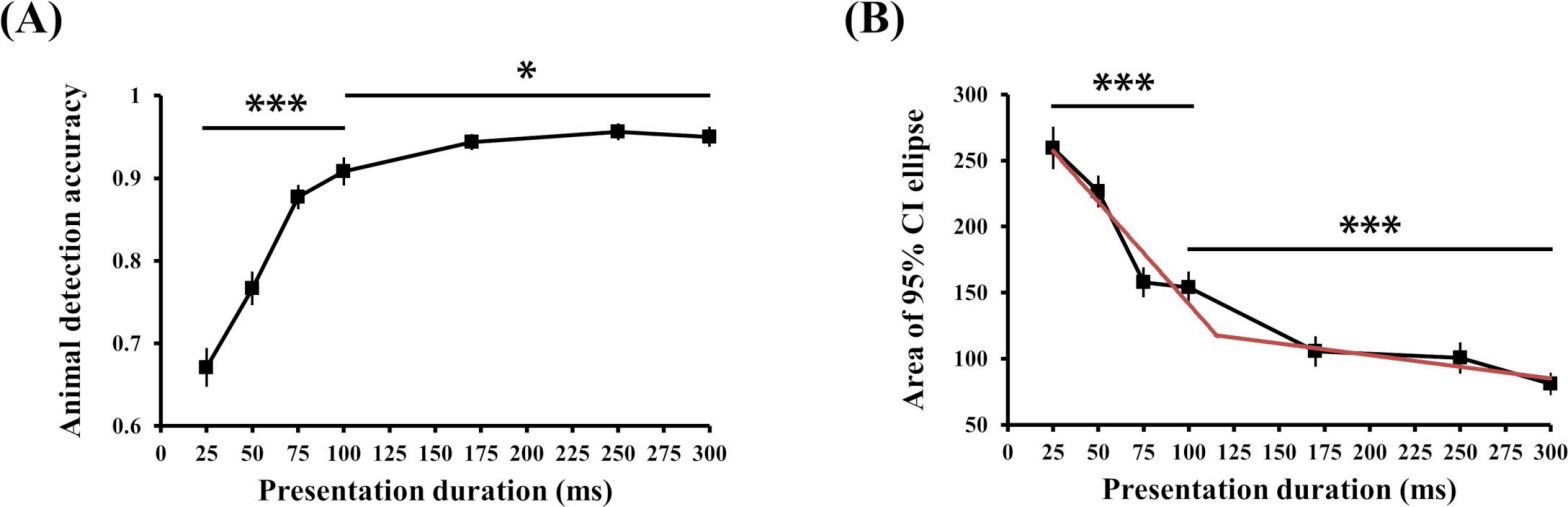
Experiment 2 results. (A) Animal detection accuracy sharply improved from 25 ms to 100 ms and then it gradually improved as the stimuli were presented longer. (B) Black line: Feature localization error sharply decreased from 25 ms to 100 ms and it slowly but continuously decreased afterwards, suggesting that feedback processing enhanced localization performance. Red line: Piecewise linear fit to the average data. Error bars indicate SEM. * *p* < .05, *** *p* < .001.

Mean animal detection accuracy significantly varied depending on stimulus presentation duration (Fig 4A; Greenhouse-Geisser correction (ε = .589); *F*(3.54, 81.32) = 65.963, *p* < .001). Accuracy sharply improved between 25 ms and 100 ms (*F*(3, 69) = 51.013, *p* < .001), once again supporting the important role of feed-forward processing in object categorization. Nevertheless, performance also improved gradually between 100 ms and 300 ms (Greenhouse-Geisser correction (ε = .69); *F*(2.07, 47.59) = 4.532, *p =* .015). Across the two experiments, mean animal detection accuracy at the shortest presentation duration decreased from 84.33% (SD 11.32%, at 20 ms in the *before* condition) in Exp 1 to 68.33% (SD 10.98%, at 25 ms) in Exp 2. This indicated that rapid object categorization is slowed by increasing image complexity [49–52] and that top-down feedback processing could help segmenting the target object category in complex scenes.

#### Feature localization

The presentation duration significantly modulated localization error (*F*(6, 138) = 36.148, *p* < .001). Mean localization error was significantly reduced from 25 ms to 100 ms (*F*(3, 69) = 16.772, *p* < .001) and was continuously reduced afterwards (100 ms to 300 ms: *F*(3, 69) = 11.393, *p* < .001). The black line in Fig 4B shows these results. This decrement in localization error across longer presentation durations was not observed in Experiment 1 which suggests that precise feature localization among several distractors requires additional processing time, and thus, involves top-down feedback processing. Nevertheless, the present results might be driven by the addition of a longer presentation duration (300 ms). Therefore, we analyzed mean localization errors from 100 ms to 250 ms to match the results from Experiment 1. Even after excluding the localization error at 300 ms, localization errors significantly decreased as presentation duration increased above 100 ms (*F*(2, 46) = 8.647, *p* = .001). Also, the post-hoc comparisons showed that the decrement was significant when presentation duration was extended from 100 ms to 170 ms (M_diff_ = 48.33, SE = 13.55, *t*(23) = 3.567, *p* = .002) and from 100 ms to 250 ms (M_diff_ = 53.11, SE = 15.35, *t*(23) = 3.459, *p* = .002).

Our results suggest that recurrent processing contributes to improved localization accuracy that occurs beyond, and is complementary to, the localization afforded by a feed-forward process. This is a necessity, since a single neuron in human visual area TO, for example, can only localize to the size of its RF (about 20° of visual angle at 10° eccentricity [17]). The question is how a finer spatial location is determined at the level of description afforded by that layer of neurons. Indeed, the decrement in mean localization error was much greater within the 25–100 ms range (M_diff_ = 105.72) than within the 100–300 ms range (M_diff_ = 72.82). It is likely that initial feed-forward processing enables a fast but coarse location estimation, whereas subsequent feedback processing helps a slow but more precise localization process. If the visual input is relatively simple like the stimuli used in Experiment 1, which produces little conflict within feed-forward convergence neural communication, feature localization could be completed within the feed-forward sweep. In more complex visual scenes, feedback processes would be needed to provide fine localization. As feed-forward and feedback processing have different time courses, we might be able to reveal them by fitting our data in a piecewise linear manner, having 100 ms as the breakpoint (based on the results in Exp 1) and comparing it to the linear polynomial model.

The equation of the linear polynomial fit was

y=−0.5767x+234.9

and the equations of the piecewise linear fit was when *x*<100 (ms)

y=−1.544x+295.9

when *x*≥100 (ms)

y=−0.1764x+138

To compare the goodness-of-fit of the piecewise linear model with that of the linear polynomial model (which assumes single process in localization), we computed the Akaike information criterion (AIC, [53]) which penalizes model complexity. Smaller AIC values indicate better fit. The piecewise linear model (AIC = 43.02) explained the data better than the linear polynomial model (AIC = 50.12), and the red line in Fig 4B shows the result of the piecewise linear fitting. This supports the idea that both feed-forward and feedback processing contribute to feature localization, but each operates at different time points and with different effectiveness (e.g., slopes). It also suggests that feedback processing might be necessary specifically when a localization task requires attentional engagement (e.g., filtering out irrelevant distractors).

## Discussion

The present study examined the roles of feed-forward and feedback visual processing in object categorization and localization. As many previous studies have demonstrated, the present study suggests that human vision can very rapidly determine the category of a certain object (i.e., animal) embedded in a visual scene, demonstrating that animal detection accuracy dramatically improved as stimulus presentation duration increased ~100 ms. However, animal detection accuracy at longer presentation durations was dependent on scene complexity. Performance did not significantly improve after 100 ms stimulus exposure for simple stimuli (Experiment 1), whereas it did improve at longer presentation durations when stimuli were more complex (Experiment 2). Therefore, rapid object categorization may not solely rely on feed-forward processing, but top-down feedback processing may be also involved when visual scenes need to be analyzed in more detail. Similarly, processing time for animal feature localization was dependent upon the complexity of visual scenes. Feature localization was as fast as animal detection when scenes were simple with a single animal. When scenes were cluttered, localization error was gradually reduced with increasing presentation times, indicating that feedback processing may be necessary for fine-grained localization.

The Selective Tuning model has claimed that different visual tasks require different processing strategies, such as feed-forward or feedback [34,45]. While simple tasks, including object discrimination or categorization could be achieved within a single feed-forward pass, more complex tasks that require high-resolution information (e.g., precise localization) should involve top-down feedback processing, taking additional processing time. As previously mentioned, location information is blurred in high-order areas due to the integration of earlier neurons’ RFs and because diverging feed-forward connections along the visual processing hierarchy cause cross-talk among visual signals. Hence, a top-down traversal that ameliorates this cross-talk must reach early visual areas where the finest location information is available [54]. An important consequence of top-down localization is that when it selects target input connections, the remainder of the input (i.e., noise) within the same RF is suppressed, forming a suppressive surround around the target which eventually enhances the overall signal-to-noise ratio of the neuron [20,35,37,55–57]. Thus, the manifestation of the suppressive surround indirectly supports a top-down localization mechanism. Experimentally, it has been reported that a visual task that requires precise feature-location binding produces a suppressive surround but not a simple discrimination task. Furthermore, the effect of the surround suppression becomes evident around at 250 ms after stimulus onset [35]. This delay relative to the time course of the initial feed-forward sweep is consistent with the temporal range of top-down attentional modulation in early visual cortex [38,58–62], implying that fine-grained localization completes within this feedback pass.

We used different scene complexities across the experiments and each led to different results in the categorization and localization tasks. In Experiment 1, target animals were centered and occupied large portions of images. Their backgrounds were also simple so that targets could be segregated from them easily. Due to these factors, competitive top-down selection of object information might not have been necessary for categorization and localization. Thus, both tasks were achieved within the feed-forward time range. In the localization task, it is possible that participants had localized the features based on the coarse layout of the animals and the relative spatial locations of the features without knowing their actual locations. ST suggested that this coarse level of location details is provided at intermediate layers of processing hierarchy, so the localization task in Experiment 1 is unlikely to necessitate a full top-down feedback traversal for more precise location information. It is also consistent with the findings that coarse location information is available in intermediate or higher-order object selective areas [21–29] (but see also [63]), allowing rapid access to this information after a single feed-forward sweep. On the other hand, more complex stimuli were used in Experiment 2 wherein animals were often presented with other distracting objects (e.g., humans), and the target animal was embedded in a complex and realistic background. These stimuli make target-background segregation much more difficult. As a result, task difficulty increased and both categorization and localization performance improved when additional processing time was provided, consistent with the contribution of feedback processing. Previous studies have reported that rapid object categorization is impaired when target objects are embedded in a complex background rather than in a simple background [49,51,52], and that categorization is attention-dependent when multiple foreground objects are presented together [50]. The results of the present study are in line with these findings and they further suggest that object categorization in complex visual scenes requires time-consuming, top-down feedback processing. Similarly, feature localization in complex scenes requires a top-down, attention-mediated selective mechanism to overcome the crosstalk within the visual hierarchy and thus select the targets among various distractors, as ST claims. Experiment 2 would represent the nature of fine-grained localization with top-down feedback processing better than Experiment 1 because its stimuli are inherently attention-demanding, in the similar way that feature conjunction visual search tasks are more attention-demanding than feature pop-out tasks. Moreover, they are much closer to the visual scenes we encounter in daily life, so they are more ecologically valid. Therefore, the results of Experiment 2 are likely to demonstrate the time course of precise localization more accurately.

Other theories prescribe computational decoding procedures that can take high level cortical representations as input and decode them to extract meaning, in particular, location information. For example, Hung et al. [14] used a classifier-based readout technique to interpret the neural coding of selectivity and invariance at the IT population level. The activity of small neuronal populations over very short time intervals (as small as 12.5 ms) contained accurate and robust information about both object identity and category. Coarse information about position and scale could be read out over three positions. Isik et al. [15] used neural decoding analysis to understand the timing of invariant object recognition in humans. They found that size and position-invariant visual information appears around 125 and 150 ms, respectively, and both develop in stages, with invariance to smaller transformations arising before invariance to larger transformations. They claimed that this supports a feed-forward hierarchical model of invariant object recognition where invariance increases at each successive visual area along the ventral stream. This is in contrast to work by Zhang et al. [64] who showed how attention influences object position and identity information represented by the population of IT neurons when there is competition among objects (i.e., cluttered display). They found that before attention was employed, visual clutter significantly reduced the object information relative to when single object was presented. However, when attention was directed to a specific object, the amount of object information was restored to nearly the same level when the object was shown in isolation.

The difference between the results in these last two papers is due to the different stimuli used, the latter requiring attention and the former not. We can conclude that although coarse location information is likely easily extracted after a single feed-forward pass for simple recognition tasks, more complex visual tasks that require image details (e.g., precise feature location) likely are not. Something more is needed for natural images and for tasks where more precision is required than simple coarse position [54]. There are really two choices: 1) provide mechanisms that dynamically ameliorate the interference before interpretation; or, 2) provide mechanisms to correctly interpret corrupted representations. The methods just described are of the latter type. ST advocates for the former possibility and our experiments provide evidence to support this.

Another explanation could be that precise location simply emerges over time from the results of feed-forward processing, perhaps using some kind of evidence accumulation mechanism, and that no top-down process is at play at all. If this were true, there would be no recurrent suppressive surround or any kind of backwards activations throughout the cortex, as has been observed in other studies [35,54,65,66]. In the emergence explanation, brain imaging methods would only observe increasing activation over time within one area and this is not the case. This would simply produce a single linear process for the increase in localization precision with increasing time, whereas Experiment 2 supported dual processes, a feed-forward process as well as a recurrent process. One might argue that fast local recurrent feedback within the ventral pathway of the visual system could achieve tasks that require visual scrutiny since mounting evidence supports that local feedback may compensate for disrupted initial feed-forward signals [39,67–69]. For example, a recent MEG-fMRI study [40] demonstrated recurrent activity from IT to early visual cortex during object categorization that they did not attribute to attentional modulation due to the relatively early emergence of the feedback signal compared to the typically reported attention-related top-down signal [35,38,39]. However, the visual task they used is a simple face detection task that probably does not require precise feature binding nor localization, and the authors also acknowledged that local recurrent activity is involuntary and independent of attentional modulation [70,71]. Therefore, that study might not test the role of top-down feedback processing in object recognition as local recurrent activity is unlikely to complete attention-demanding recognition tasks on its own.

To conclude, the current study demonstrates that precise localization information seems to require subsequent top-down feedback processing. Other natural tasks such as comparison (are two objects the same or different?) or measurement (which object is furthest away?) may also require more detailed localization processes than are available after a single feed-forward pass. In natural environments, precise localization is critical for goal-directed behavior, such as reaching and pointing, to correctly select a target object and avoid other distracting objects [72], supporting the need for recurrent processing. It remains an open question as to how localization is involved in interaction with real-world, 3-dimensional objects. Future studies using real-world objects or virtual reality would allow greater insight into how top-down localization operates in a natural context and expand our understanding of the relationship between visual attention and action.
